# Phytomedicines Targeting Cancer Stem Cells: Therapeutic Opportunities and Prospects for Pharmaceutical Development

**DOI:** 10.3390/ph14070676

**Published:** 2021-07-15

**Authors:** Piyush Kumar Gupta, Mrunmayee Saraff, Rekha Gahtori, Nidhi Negi, Surya Kant Tripathi, Jatin Kumar, Sanjay Kumar, Saad Hamad Aldhayan, Sugapriya Dhanasekaran, Mosleh Mohammad Abomughaid, Kamal Dua, Rohit Gundamaraju, Shreesh Ojha, Janne Ruokolainen, Niraj Kumar Jha, Kavindra Kumar Kesari

**Affiliations:** 1 Department of Life Science, School of Basic Sciences and Research (SBSR), Sharda University, Knowledge Park III, Greater Noida 201310, Uttar Pradesh, India; jatin.kumar1@sharda.ac.in (J.K.); sanjay.kumar7@sharda.ac.in (S.K.); 2 Department of Microbiology, St. Xavier’s College (Autonomous), Mumbai 400001, Maharashtra, India; mrunmayeesaraff@gmail.com; 3 Department of Biotechnology, Sir J. C. Bose Technical Campus, Kumaun University, Nainital 263136, Uttarakhand, India; rekhagahtori11@gmail.com; 4 Department of Chemistry, DSB Campus, Kumaun University, Nainital 263001, Uttarakhand, India; nidhi.negi2@gmail.com; 5 Cancer Drug Resistance Laboratory, Department of Life Science, National Institute of Technology, Rourkela 769008, Odisha, India; suryabiotech1309@gmail.com; 6 Pharmaceutical Department, Prince Sultan Military Medical City, Riyadh 12233, Saudi Arabia; Saldhayan@psmmc.med.sa; 7 Medical Laboratory Sciences Department, College of Applied Medical Sciences, University of Bisha, Bisha 67714, Saudi Arabia; sughaphd@yahoo.com (S.D.); moslehali@ub.edu.sa (M.M.A.); 8 Discipline of Pharmacy, Graduate School of Health, University of Technology Sydney, Sydney, NSW 2007, Australia; Kamal.Dua@uts.edu.au; 9 ER Stress and Mucosal Immunology Laboratory, School of Health Sciences, University of Tasmania, Launceston, TAS 7248, Australia; rohit.gundamaraju@utas.edu.au; 10 Department of Pharmacology and Therapeutics, College of Medicine and Health Sciences, P.O. Box 17666, United Arab Emirates University, Al Ain 15551, United Arab Emirates; shreeshojha@uaeu.ac.ae; 11 Department of Applied Physics, School of Science, Aalto University, 00076 Espoo, Finland; janne.ruokolainen@aalto.fi; 12 Department of Biotechnology, School of Engineering & Technology (SET), Sharda University, Greater Noida 201310, Uttar Pradesh, India; nirajkumarjha2011@gmail.com; 13 Department of Bioproducts and Biosystems, School of Chemical Engineering, Aalto University, 00076 Espoo, Finland

**Keywords:** phytomedicines, phytochemicals, stem cells, signaling pathway, epithelial–mesenchymal transition, preclinical, clinical research

## Abstract

The presence of small subpopulations of cells within tumor cells are known as cancer stem cells (CSCs). These cells have been the reason for metastasis, resistance with chemotherapy or radiotherapy, and tumor relapse in several types of cancers. CSCs underwent to epithelial–mesenchymal transition (EMT) and resulted in the development of aggressive tumors. CSCs have potential to modulate numerous signaling pathways including Wnt, Hh, and Notch, therefore increasing the stem-like characteristics of cancer cells. The raised expression of drug efflux pump and suppression of apoptosis has shown increased resistance with anti-cancer drugs. Among many agents which were shown to modulate these, the plant-derived bioactive agents appear to modulate these key regulators and were shown to remove CSCs. This review aims to comprehensively scrutinize the preclinical and clinical studies demonstrating the effects of phytocompounds on CSCs isolated from various tumors. Based on the available convincing literature from preclinical studies, with some clinical data, it is apparent that selective targeting of CSCs with plants, plant preparations, and plant-derived bioactive compounds, termed phytochemicals, may be a promising strategy for the treatment of relapsed cancers.

## 1. Introduction

Cancer is one of the deadly diseases affecting the population worldwide, despite advancements in numerous therapeutic interventions. One of the major problems in cancer treatment is drug resistance, and cancer stem cells (CSCs) have been found as one of the important mediators to impart resistance [1,2]. CSCs are the drug-resistant cells that possess a unique ability for self-renewal, which makes them immortal [3]. There are numerous pluripotency-associated transcription factors, such as Oct4, Sox2, and Nanog, which play an essential role in maintaining the stemness of these CSCs [4]. Due to the stemness, CSCs lead to tumor heterogeneity and aggressiveness, which eventually results in metastasis [5]. CSCs also impart the dormancy of the tumors that causes treatment resistance and increases the chance of relapse [6]. CSCs are responsible for the initiation and progression of cancer as well as recurrence after treatment. Thus, CSCs have generated interest in understanding cancer treatment and prognosis in recent years [7,8]. CSCs account for EMT, which makes the cells more motile and invasive. Aberration or dysregulation of various molecular and cellular signaling pathways as well as altered metabolism of CSCs and dysregulated EMT further exacerbate the tumor heterogeneity. Altogether, the evidence points out that CSCs play a crucial role in cancer dissemination, from initiation to progression and relapse [9].

Since cancer is a fatal disease affecting millions of people worldwide, there is a great necessity of different treatment options to overcome the drug resistance or recurrence conditions in cancer patients. For the treatment of cancer, the conventional modalities are radiotherapy and chemotherapy. However, in recent years, accumulating experimental and epidemiologic studies have demonstrated that the plants and plant-derived bioactive agents, popularly known as phytochemicals, showed medicinal value, and appear beneficial as chemo-preventive and chemotherapeutic agents. Medicinal plants and their bioactive compounds have been a very good and easily accessible source for the development of novel therapeutics for various cancer diseases. Many of them have also been found to exert chemo-sensitizing effects and synergize the anticancer effects, and thus may be useful in drug-resistant cancer cells. Collectively, the plant-based formulations available either as a single herb formulation, or a mixture of many plant extracts or the plant-derived compounds, are called phytomedicines, and these have attracted interest to be future candidates in cancer therapy, owing to their selective cytotoxicity against cancer cells as well as fewer or negligible adverse effects [2]. Phytomedicines exist either in isolated or purified form or as a mixture of different secondary metabolites, and are used to prevent and cure different diseases [10]. Phytomedicines may also have vitamins and minerals which are believed to synergize preventive and therapeutic effects, and additionally be useful in treatment of drug-resistant cancers [10]. The plant extracts or plant-derived bioactive constituents have been tested for several years and showed anti-tumor activity by modulating the dysregulated signaling pathways, targeting efflux pump or transporters, and/or inducing apoptotic cell death and cell cycle arrest. Even though several purified bioactive phytocompounds and crude extracts of thousands of medicinal plants have been tested for their therapeutic effects during cancer disease treatment, very few of them have been studied on both in vitro and in vivo platforms, and only a few of them are under clinical trials.

In recent years, the utilization of many plant extracts and plant-derived agents has gained momentum for their activity against CSCs. The available studies are indicative of their anti-CSC properties mediating the modulation of numerous signaling pathways, which participate in the physiological and molecular regulation of CSCs. Many studies have shown that medicinal plants, plant-derived bioactive compounds, or the plant formulations commonly used in traditional Chinese medicines (TCM) reduce the stem-like characteristics of CSCs. They exhibit their activities by interfering with EMT genes, reducing invasiveness, and inhibiting migratory properties of CSCs [11]. In purview of the increasing understanding of the role of CSCs in many cancer types, in the present review, we comprehensively discussed the recent studies showing targeting of the CSCs by phytomedicines. The mechanisms and effects are presented in synoptic tables and schemes. The present review is suggestive of the therapeutic opportunities and prospects of phytomedicines and encouraging further studies for their pharmaceutical development.

## 2. Cancer Stem Cells and Their Markers

**Figure 1:**
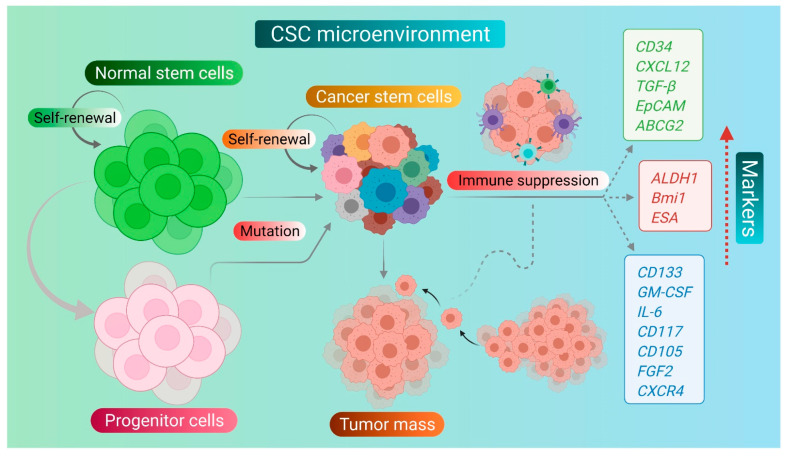
CSCs and their overexpressed biomarkers.

The process of tumorigenesis has been explained by two different models, namely the stochastic model and the hierarchical model, also known as the CSC model. According to the stochastic model, the transformation of somatic cells leads to the generation of tumors. In contrast, the hierarchical model states that CSCs are the mainstay of the tumor origin and growth [12,13]. In general, the CSCs are derived clonally by multiple symmetric or asymmetric cell divisions of cancer progenitor cells (CPCs) or transformed stem cells [8]. Further, the CSCs lead to the development of the aggressive or relapsed form of metastasizing tumors. CSCs are also known to express certain specific antigens which act as molecular biomarkers and help in their validation and identification, as illustrated in Figure 1. These overexpressed biomarkers are often employed to characterize and isolate different types of CSCs from drug-resistant cancer cell populations [14,15]. Further, these biomarkers of the CSCs are also utilized as a target to develop novel therapeutic targeted therapies, as summarized in Table 1.

Tumor cells are known to undergo phenotypic alteration as a consequence of EMT during cancer progression. In EMT, the epithelial cells develop the traits of mesenchymal cells, which are characterized by downregulation of E-cadherin and upregulation of N-cadherin mediated by numerous transcription factors such as Snail, Slug, and Twist [27,28]. Tumor cells under EMT undergo enhanced motility and migration properties [29]. Additionally, when tumor cells undergo metastasis, they possess through five orchestrated steps, such as invasion, intravasation, transport, extravasation, and colonization [30]. EMT is required for the intravasation and extravasation of tumor cells, but its loss is also eventually needed to achieve the proliferation of tumor cells. The reversal of the EMT process, termed mesenchymal–epithelial transition (MET), helps in tumor proliferation and growth [30,31]. Due to such transition process, tumor cells become more invasive, gain capability to metastasize, and impart resistance to the chemotherapy and radiotherapy in cancer treatment [32].

The regulation of CSCs is carried by several different signaling mechanisms, such as Janus-activated kinase/signal transducer and activator of transcription (JAK/STAT), Nuclear factor-kappa B (NF-κB), phosphatidylinositol 3-kinase (PI3K)/protein kinase B (Akt) (PI3K-Akt), Hedgehog (Hh) pathway, Wnt/β-catenin, and Notch pathways [14,33]. These cellular signaling pathways have been shown to mediate the stemness of CSCs, which are discussed briefly below.

### 2.1. JAK/STAT Pathway

The JAK/STAT pathway is one of the important pleiotropic signaling pathways which play a vital role in transmission of signals from cell-membrane receptors to the nucleus and contribute to the immune-inflammatory mechanisms mediating cytokines and growth factors. The ligands such as interleukins, growth factors, or hormones bind to the receptors and bring together two associated JAKs that facilitate the phosphorylation of each other on tyrosines, and become fully activated. Consequently, they phosphorylate the receptors and generate binding sites for STAT proteins. Further, the JAKs phosphorylate the STAT proteins, which dissociate from the receptor to form dimers and enter into the nucleus to regulate the gene expression. The overexpression of several genes such as IL-6 and CSF2 as well as highly activated STAT1 or STAT3 constitute a check on the aberration of this pathway in CSCs [34].

### 2.2. PI3K-Akt Pathway

The PI3K/Akt signaling pathway plays an important role in the regulation of physiological processes and controls cell survival and proliferation by checking cell cycle, growth, metabolism, proliferation, growth, and angiogenesis. Overactivation of this intracellular pathway has been demonstrated to play a crucial role in numerous cancer types. Mechanistically, when ligands bind to the receptor tyrosine kinases, then plasma membrane-bound enzyme PI3K is activated and converts phosphatidylinositol (3,4)-bis-phosphate (PIP2) to phosphatidylinositol (3,4,5)-trisphosphate (PIP3). PIP3 acts as a docking site for protein kinase B (PKB), also called Akt. Further, PKB undergoes phosphorylation and is activated by mammalian target of rapamycin (mTOR) and the phosphoinositide-dependent kinase 1 (PDK1). The activated PKB inhibits the apoptosis by phosphorylating Bad. PTEN, a phosphatase, acts as a negative regulator of the process, causing the dephosphorylation of PIP3 to PIP2. Additionally, the constitutive activation of PKB or inactivation of PTEN has been observed as a reason for tumor generation in various cancers [35].

### 2.3. NF-κB Pathway

The NF-κB pathway, the most conserved and well-studied master regulator of innate immunity, exists in an inactive form in the cytoplasm. It is known to play complex roles in linking pathogenic signals and cellular danger signals and can be either canonical or non-canonical in nature. It has been shown to play an important role in regulating the transcription of DNA, cytokine production, and cell survival, and is crucial in mitochondrial function and dysfunction. In the canonical NF-κB pathway, the binding of ligands (IL-1β or the components derived from bacterial cell wall) to their respective receptors (IL-1 receptor or toll-like receptors) causes the recruitment of adaptor proteins, which in turn causes phosphorylation of IkB, making it available for the ubiquitination and proteasome degradation. As a result, NF-κB is released, which translocates to the nucleus and facilitates the gene transcription. Whereas, in the noncanonical NF-κB pathway, it involves activation by the receptor activator of NF-κB (RANK) and CD40. Thereafter, the kinases ensue the phosphorylation and process p100/RelB dimers into p52/RelB dimers. Consequently, NF-κB is released and translocates into the nucleus, where it facilitates the transcription [36].

### 2.4. Hedgehog Pathway

The Hh pathway is a relatively recent signaling cascade that has been identified to play an important role in many processes, including embryonic development and tissue homeostasis. The mammalian cells have three hedgehog homologues, including Sonic Hedgehog (SHh), Indian Hedgehog (IHh), and Desert Hedgehog (DHh). When these homologues interact with the target cells, they bind with Patched 1 (PTCH) cognate receptors involved in this pathway. When the receptors are unoccupied by the ligands, then it acts as a constitutive inhibitor of a transmembrane protein, i.e., Smoothened (Smo). Further, the transcription of the target gene is repressed by the Gli repressor. When ligands occupy the Patched 1 receptor, it causes the release of Smo and allows the Gli transcriptional activators to enhance the transcription of target genes [37].

### 2.5. Wnt/β-Catenin Pathway

The Wnt/β-catenin pathway comprises of a group of signal transduction pathways that start with proteins passing cellular signals, either in the closest cell-to-cell communication (paracrine) or communication within the same cell (autocrine). The Wnt pathway comprises of canonical (β-catenin-dependent) and non-canonical (β-catenin-independent) signaling pathways. The canonical pathway is of paramount importance due to its role in survival of the CSCs. In the canonical pathway, β-catenin phosphorylates in the absence of Wnt ligands (Wnt3a and Wnt1) following its interaction with the destruction complex, which consists of the scaffold proteins, Axin, APC, GSK3β kinase, and casein kinase (CK1α). This phosphorylation brings the ubiquitination and degradation of β-catenin. On the other hand, when Wnt binds to the frizzled (Fzd) receptors and/or the low-density lipoprotein-related protein (LRP) co-receptors, it results in activation of this pathway. Consequently, disheveled (Dvl) proteins are recruited that inactivate the destruction complex and result in stabilization and accumulation of β-catenin. This further translocates into the nucleus, and binds to the lymphoid enhancer factor (LEF)/T-cell factor (TCF) and facilitates the transcription of various target genes [38].

### 2.6. Notch Pathway

The Notch signaling pathway, a well-conserved cell signaling pathway, mediates juxtracrine cellular signaling by regulating cell fate decisions and tissue differentiation in neuronal, cardiac, immune, and endocrine tissues in embryonic development, and maintains homeostasis. The Notch pathway comprises of different types of Notch receptors, such as Notch1, Notch2, Notch3, and Notch4. The interaction and binding of Notch ligands (Delta-like-1, DLL3, DLL4, Jagged1, or JAG2) with NOTCH receptors (Notch1–4), ADAM/TACE, and γ-secretase in a sequential manner commence the proteolytic cleavage of the cytoplasmic domain of the receptor. This dual cleavage results in the release of Notch intracellular domain (NICD) into the cytoplasm. Then, it translocates into the nucleus and activates the transcription of target genes via the CBF1, suppressor of hairless (Su(H)), and LAG-1/recombining binding protein J-kappa (CSL/RBPJ) transcription factors [39].

## 3. Phytomedicines Targeting Key Regulators of Anti-Cancer Drug Resistance in CSCs

**Figure 2:**
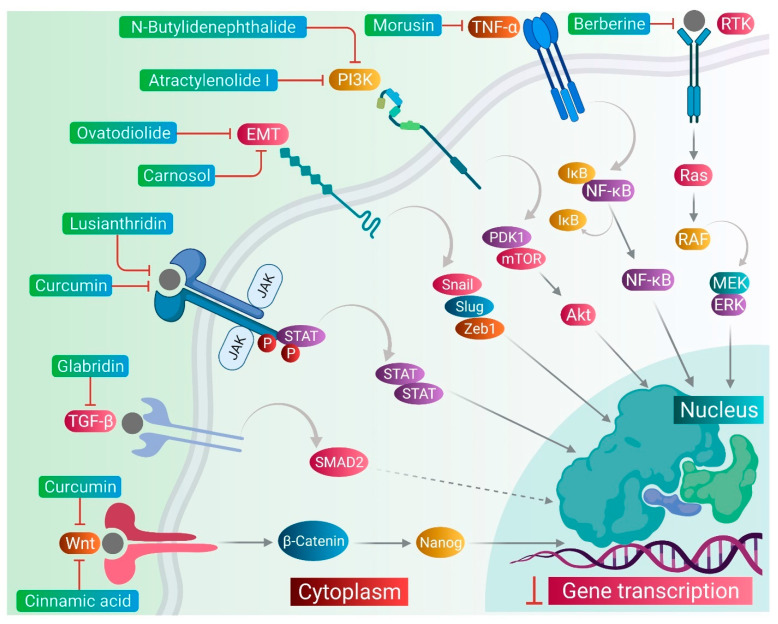
Phytomedicinal compounds targeting the key regulators of anti-cancer drug resistance in CSCs, such as Curcumin/Cinnamic acid: Wnt; Glabridin: SMAD2; Curcumin/Ovatodiolide/Lusianthridin: JAK/STAT; Ovatodiolide/Carnosol/N-butylidenephthalide: EMT; N-butylidenephthalide/Atractylenolide I: PI3K/Akt; Morusin: NF-κB; Berberine: Ras/RAF.

**Figure 3:**
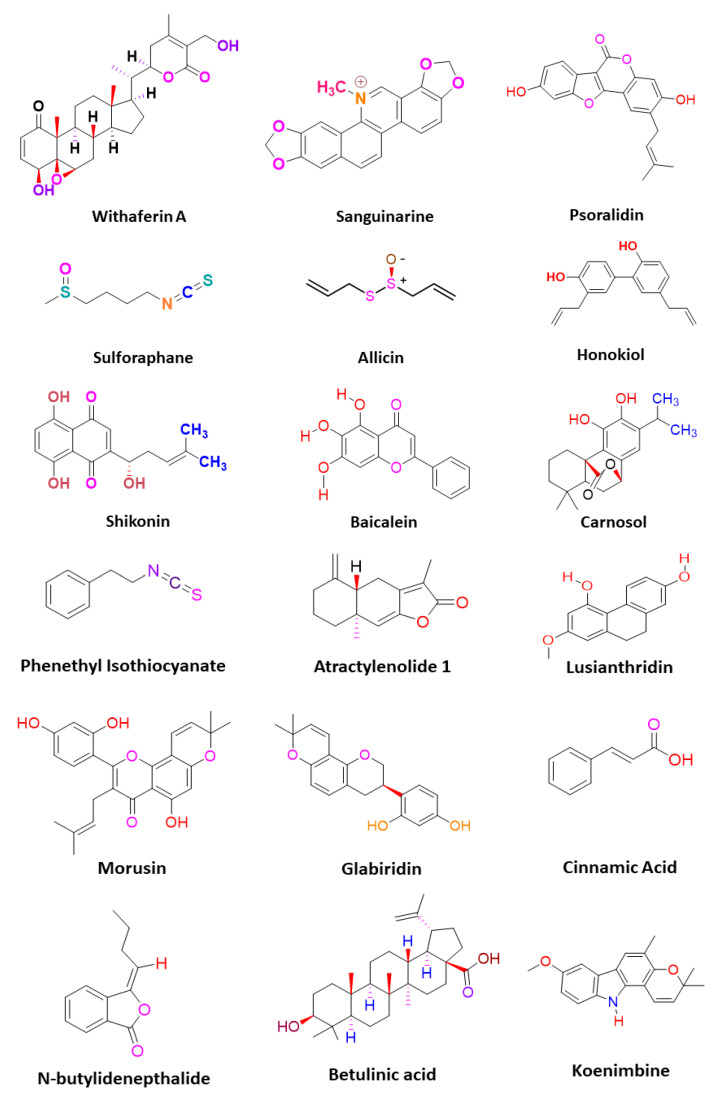

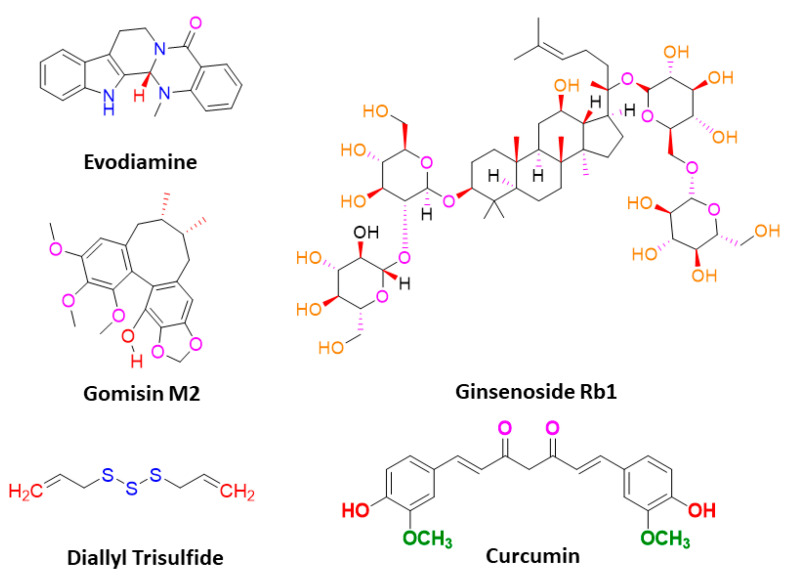
Phytomedicinal compounds targeting different key regulators of anti-cancer drug resistance in CSCs.

CSCs contribute to the anticancer drug resistance by numerous mechanisms, including EMT regulation, enhanced expression of ABC transporters, overexpression of aldehyde dehydrogenase (ALDH) enzyme, slow cycling of microRNAs, regulation of tumor microenvironment, as well as resistance to DNA damage and cell death [15]. Phytomedicines target any one of these key regulating machineries in the resistance of CSCs to the anticancer drugs (Figure 2). The targeting of these mechanisms could be important in ceasing or eliminating CSCs and it may improve the outcome of anticancer drugs during chemotherapy of cancer. Some of these phytomedicines are discussed in this section and their related information and figures are provided in Figure 3 and summarized in Table 2.

Curcumin, a polyphenol from rhizomes of turmeric (*Curcuma longa*), a popular dietary component, is one of the highly studied and regarded natural agents for numerous biological properties, including potent anticancer, chemo-preventive, and chemo-sensitizing activities, and benefits in synergizing anticancer activity and reducing dose-limiting organ toxicity. Recently, many reports revealed that curcumin targets CSCs in cancers of breast, thyroid, and brain. Curcumin was shown to act in many ways and appeared to be a polypharmacological agent in modulating numerous signaling pathways and transcription factors. All these mechanisms finally converge in reducing the tumor cells. In one study, curcumin downregulated EMT (vimentin, fibronectin, β-catenin) and stemness markers (Sox2, Nanog, Oct4) [47]. In another study, it reduced the overexpression of ABC transporters in breast CSCs [48]. In papillary thyroid CSCs, CUR dysregulated the JAK/STAT3 signaling pathway [49]. One of the major barriers in pharmaceutical development of curcumin is its bioavailability. Thus, in order to achieve the better stability, good aqueous solubility, and bioavailability of CUR, many novel drug delivery systems have been developed and are underway for further evaluation [50,59,60]. Correspondingly, the liposomal curcumin showed enhanced permeability and strong anticancer therapeutic efficacy for various drug-resistant cancers, including glioblastoma [50]. Next, Ovatodiolide (Ova) is a macrocyclic diterpenoid, isolated from *Anisomeles indica*. It exhibited potent anticancer actions on glioblastoma, nasopharyngeal carcinoma, and oral cancer cells [23,24,26]. Mechanistically, Ova reduced the stemness markers (CD44, CD133, Sox2, Klf4, Nanog, and Oct4) of CSCs and decreased the expression of EMT genes [23,24]. It also modulated the JAK2/STAT3 signaling pathway by inhibiting either JAK2 or STAT3 protein, and thus dysregulated the gene transcription [24]. In addition, Ova induced apoptotic cell death and exerted cytotoxicity of the cancer cells. The findings were further translated in an in vivo study, in an oral carcinoma (SAS cells) xenograft mice model, wherein Ova (3.6 mg/kg) reduced tumor growth, 2.2-folds less, compared to the untreated mice [24].

Lusianthridin, a phenanthrene derivative and phenolic compound isolated from the stem of the plant *Dendrobium venustum,* was shown to downregulate the Src-STAT3-c-Myc signaling pathway and suppress CD133, ABCG2, and ALDH1A1 stemness markers, which induced the apoptosis in lung CSCs [22]. The root extract of *Polygonum cuspidatum* consisting of 2-ethoxystypandrone, i.e., a novel analogue of juglone, was shown to exhibit inhibition of the STAT3 signaling pathway in hepatocellular carcinoma cells (HCC cells). It ceased the growth and proliferation of HCC cells in a dose-dependent manner and induced the programmed cell death of CSCs in HCC [53].

Although a majority of the phytomedicines have been shown to target the cell death or apoptosis pathway, a few of them were also shown to target the cell cycle arrest, such as two monoterpenoid indole alkaloids, Scholarisine Q(1) and R(2), isolated from the fruit extract of *Alstonia scholaris,* which were found to induce apoptosis in glioma stem cells [40]. Another phytoconstituent, berberine, an isoquinoline alkaloid compound abundantly found in the plant *Berberis vulgaris,* was found to be effective in reducing the stemness, cell migration, and cell growth (*via* G0–G1 cell cycle arrest) of neuroblastoma and prostate CSCs, respectively [32,46]. Similarly, extract of *Viola odorata* rich in numerous bioactive components, such as saponin, salicylic acid derivatives, glycosides, alkaloids, anthocyanidins, and cyclotides, was shown to induce apoptosis and reduce the growth and migration of breast CSCs [52].

Carnosol, a polyphenolic diterpene abundantly found in *Rosmarinus officinalis,* showed modulation of the EMT genes and induced apoptosis in glioblastoma CSCs [57]. Phenethyl isothiocyanate (PEITC), a natural isothiocyanate predominantly present in the cruciferous vegetables such as broccoli and watercress, was shown to enhance oxidative stress in CSCs and downregulate the expression of stemness genes in various cancers, such as cervical and colon cancers [42]. In an in vivo study employing an ALDH^+^ HeLa CSCs xenografted NOD-SCID mice model, PEITC at the concentration of 10 μM exhibited a reduction in the tumor volume when compared to the untreated mice serving as control mice [42]. In another study, PEITC at the dose of 20 mg/kg suppressed the growth of EpCAM^+^ CSCs isolated from HCT116 cancer cells and also displayed a reduction in the tumor growth of a colon cancer xenograft mice model injected with EpCAM^+^ CSCs [41]. Atractylenolide-1 (ATL-1) is a sesquiterpene isolated from the rhizome extract of *Atractylodes macrocephala Koidz,* a popular Chinese medicinal herb. ATL-1 (25–75 mg/kg) downregulated the Akt/mTOR pathway and altered the glucose metabolism and stem-like behavior in colon cancer cells. It also inhibited the colorectal tumor progression in xenografted nude mice [43]. The total polyphenolic fractions obtained from *Fructus viticis* were shown to modulate the Akt/mTOR pathway and repressed the stemness characteristics in lung CSCs [44].

Cinnamic acid, a monocarboxylic acid isolated from the bark of cinnamon (*Cinnamomum zeylanicum*), has been shown to decrease the stemness of colon CSCs [19]. Shikonin, a naphthoquinone derivative abundantly found in the roots of a Chinese medicinal herb, *Lithospermum erythrorhizon,* modulated the JNK/c-Jun pathway and augmented its cytotoxicity in glioblastoma stem cells (GSCs). The findings observed in the in vitro studies were confirmed in a GSCs-xenografted mice model [56]. Morusin, a flavonoid present in the roots of *Morus australis,* was found to attenuate NF-κB activity in cervical CSCs and induced apoptotic cell death in these CSCs by curbing their migration and growth. Furthermore, this compound exerted cytotoxicity of the cervical CSCs [55]. Glabridin, an isoflavane obtained from the roots of *Glycyrrhiza glabra,* modulated epigenetic regulation of miR-148a/SMAD2 signaling and inhibited stem cell-like properties of human breast cancer cells. Additionally, Glabridin (20 mg/kg) improved the survival of breast cancer mouse xenografts [54]. Similarly, Cajaninstilbene acid derivatives of pigeon pea modulated the cytotoxicity (pathway not deduced) in breast cancer stem-like cells [45].

It has been reported that ABC transporter genes such as ABCG2 and ABCB5 often become upregulated in cancers of pancreas, breast, lung, ovary, and skin, and can be the potential targets for therapy [15]. PienTze Huang (PZH), a traditional Chinese medicine (TCM) consisting of *Moschus*, *Calculus Bovis*, Snake Gall, and *Radix Notoginseng,* showed inhibition of the mRNA levels of ABCB1 and ABCG2 transporters in HT29 side population cells (HT29 CSCs) [58]. It also suppressed the growth of colorectal CSCs in a dose-dependent manner [58].

Similar to the plant-derived bioactive compounds, plant extracts have been tested in many studies. The crude extracts prepared from whole plant or certain plant parts showed potent anticancer effects in many cancer types by targeting drug-resistant CSCs in their cell populations. For example, the bark extract of *Walsura pinnata Hassk* and the rhizome extract of *Costus speciosus* induced the apoptotic cell death in the leukemic and prostate CSCs, respectively [16,51]. In the next sections, we comprehensively discuss targeting of several stemness markers, EMT genes, and cellular signaling pathways, which could be an important therapeutic approach for the elimination of CSCs from the drug-resistant cancer cell populations, as well as to keep a check on the cell growth and proliferation, therefore ceasing the tumor growth.

## 4. Phytomedicines Targeting Wnt/β-Catenin, Notch, and Hedgehog Signaling in CSCs

**Figure 4:**
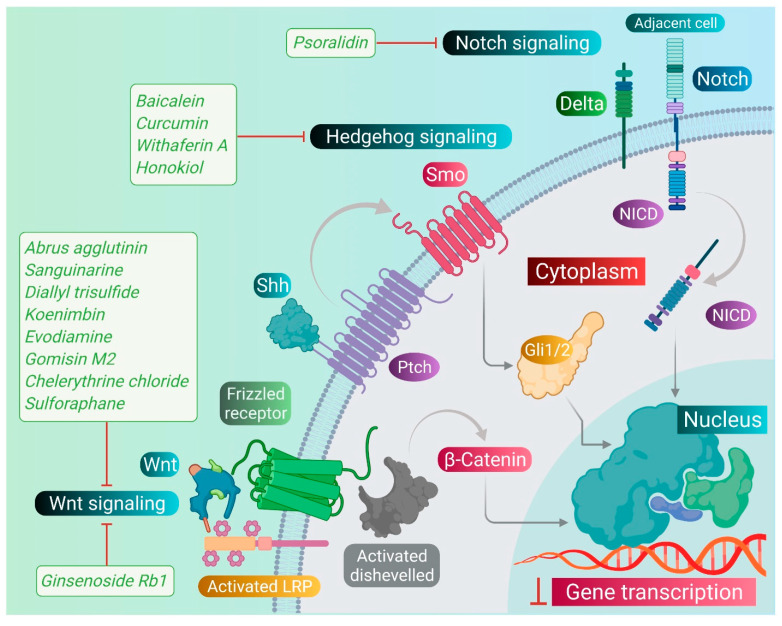
Phytomedicinal compounds targeting Wnt, Sonic Hedgehog, and Notch signaling pathways in CSCs. Abrus agglutinin, Sanguinarine, Diallyl-trisulfide, Koenimbin, Evodiamine, Gomisin M2, Chelerythrine chloride, Sulforaphane, and Ginsenoside-Rb1 inhibit the Wnt/β-catenin signaling pathway. Baicalein, Curcumin, Withaferin A, and Honokiol inhibit the Sonic Hedgehog signaling pathway, and Psoralidin inhibits the Notch signaling pathway.

As we discussed earlier, the Wnt, Notch, and Hh signaling pathways are responsible for the stem-like characteristics of cancer cells and account for their self-renewal [11]. Therefore, we primarily focused on reviewing the phytomedicines which particularly target these pathways. Therapeutic targeting of these pathways by phytomedicines could pave the design and development of natural therapeutics. Phytomedicines targeting these pathways are schematically elucidated in Figure 4.

### 4.1. Phytomedicines Targeting Wnt/β-Catenin Signaling Pathway

Phytomedicines have been shown to eliminate CSCs by modulating the Wnt/β-catenin signaling pathway. In a study, diallyl trisulfide, an organosulfur compound predominantly present in garlic, was found to enhance the expression of GSK3-β and reduce the β-catenin, signifying the suppression of the Wnt/β-catenin pathway in colorectal CSCs [61]. Likewise, Koenimbin, an alkaloidal compound extracted from the leaves of *Murraya koenigii* (L) Spreng, a plant popularly known as curry tree used in dietary preparations across Indian subcontinent and reputed for its medicinal properties, has been found to reduce the expression level of β-catenin and cyclin D1 in MCF7 CSCs. It results in suppressed formation of mammospheres and diminishes the ADH^+^ MCF7 CSC population, mediating downregulation of the Wnt/β-catenin pathway [21]. In another study, ginsenoside-Rb1 (Rb1), a natural triterpenoid saponin abundantly found in the rhizome of *Panax quinquefolius* plant and Notoginseng (a Chinese herbal medicine), was shown to target ovarian CSCs, mediating inhibition of the Wnt/β-catenin signaling pathway along with a reversal of EMT. A metabolite of Ginsenoside-Rb1, called compound K, in combination with Rb1 inhibited the self-renewal capacity of ovarian CSCs (isolated from patients) in a xenograft tumor mice model and sensitized these CSCs for their cytotoxic actions by chemotherapeutic agents, such as cisplatin and paclitaxel [62].

Abrus agglutinin, a lectin, isolated from the seeds of *Abrus precatorius,* downregulated the CD44^+^ expression in FaDu cells (oral cancer cells) and inhibited the growth and plasticity of FaDu orospheres. Further, it inhibited the Wnt/β-catenin signaling pathway and suppressed the self-renewal capacity of FaDu-derived CSCs. This compound also induced apoptosis in FaDu CSCs in a dose-dependent manner. The actions were later reconfirmed in FaDu xenografted nude mice, wherein it ceased the tumor growth [63]. Another compound, sulforaphane, isolated from the cruciferous vegetables such as broccoli and cabbage, inhibited the formation of nasopharyngeal tumor spheroids enriched with CSCs. Sulforaphane at the dose of 60 mg/kg reduced the tumor growth in C666-1 cells in a xenografted mice model through the DNA methyltransferase 1/Wnt inhibitory factor 1 axis [64]. It possesses strong anticancer activities. Treatment with sulforaphane also suppressed the expression of miR-19, which regulates the miR-19/GSK3β/β-catenin axis and the traits of lung CSCs. It also downregulated the Wnt/β-catenin pathway and β-catenin/TCF transcriptional activity in lung CSCs. Following treatment with sulforaphane, the lung tumorospheres did not develop and reduced expression markers of lung CSCs [65]. Further, Chelerythrine chloride, a benzophenanthridine alkaloid isolated from *Chelidonium majus,* downregulated the expression of Sox2, MYC, and β-catenin in SK-LU-1 and NCI-H1703 cells. This showed inhibition of the Wnt/β-catenin pathway in lung cancer cells mediating downregulation of β-catenin and resulting in curtailing the CSC properties and inducing apoptosis [66].

Sanguinarine, a benzophenanthridine alkaloid obtained from *Chelidonium majus L.* Plant (traditional Chinese medicine celandine), downregulated the Wnt/β-catenin signaling pathway and inhibited the proliferation and invasion of lung CSCs, thereby inducing the apoptosis in lung CSCs [67]. Gomisin M2, a lignan belonging to the class of hydrolysable tannins, also forms an active component of a Chinese medicine Baizuan, and inhibited the proliferation of MDA-MB-231 and HCC1806 cells. Additionally, it suppressed the self-renewal potential of breast CSCs by downregulating the Wnt/β-catenin signaling pathway. Consequently, it blocked the formation of mammospheres in breast CSCs and induced apoptosis in breast CSCs by altering mitochondrial membrane potential [68]. Furthermore, evodiamine, a natural quinolone alkaloid isolated from *Evodia rutaecarpa,* inhibited the proliferation of bulk cultured colon cancer cells and arrested cell cycle at G2/M phase, thereby inducing apoptosis in these cells. Further, this compound repressed the expression of several genes regulating the key signaling pathways such as Notch and Wnt of colon CSCs, and eliminated these cells [69]. Moreover, evodiamine also inhibited the proliferation and induced apoptosis in gastric CSCs. This compound also decreased the expression of pluripotent stem cell markers such as Bmi-1, KLF4, Sox2, and Oct4, and EMT markers such as Slug, Zeb1, Twist, and vimentin. The observations demonstrate that evodiamine exerts an inhibitory effect on the Wnt/β-catenin signaling pathway and EMT, thus suppressing the proliferation and stem cell-like properties of gastric CSCs [70].

### 4.2. Phytomedicines Targeting Notch Signaling Pathway

Like the Wnt signaling pathway, the Notch pathway also maintains the stemness of CSCs. A traditional Chinese medicine (TCM), Qingyihuaji formula (QYHJ), composed of different Chinese herbs, including *Herba Scutellariae Barbatae*, *Herba Hedyotdis*, *Herba seu Radix* *Gynostemmatis Pentaphylli*, *Rhizoma Arisaematis Erubescentis*, and *Fructus Amomi Rotundus,* was found to target the Notch signaling pathway. This formulation reduced the CD133 expression on pancreatic CSCs. Additionally, it downregulated the expression of the Notch-4 gene, but in combination with gemcitabine, it significantly suppressed expression of Notch-1, Notch-2, and Notch-3 genes, too. Furthermore, QYHJ, at the dose of 36 g/kg, inhibited tumor growth in SW1990 cells xenografted tumor mice models, suggesting that QYHJ possesses the potential to increase the survival time of patients by reducing pancreatic CSCs [71]. Similarly, another TCM formulation, Xiaotan Sanjie (XTSJ), composed of many herbs, inhibited the cell viability of gastric CSCs in a dose-dependent manner, attributed to the downregulation of Notch-1 expression, i.e., regulating the proliferation of gastric CSCs. Further, XTSJ at different doses (1.46, 2.92, and 5.84 g/mL) reduced the tumor growth in a dose-dependent manner in gastric CSC-transplanted mice models [72]. Another very popular TCM preparation, Pien Tze Huang (PZH), decreased the percentages of side population cells in SW480 cells. Additionally, it reduced the viability of side population cells in a dose-dependent manner and induced apoptosis in these side population cells, as evidenced by fragmented nucleus and condensed chromatin. Subsequently, PZH downregulated the Notch1 gene expression in colon CSCs, demonstrating its action as a potent agent targeting CSC [73]. Next, Psoralidin, a prenylated coumestans derivative isolated from the seeds of *Psoralea corylifolia,* inhibited Notch-1 signaling in breast CSCs that promoted the inhibition of EMT markers. This resulted in decreased invasion and migration of ALDH+ breast CSCs. Further, it inhibited the growth and induced programmed cell death in breast CSCs [74].

### 4.3. Phytomedicines Targeting Hedgehog Signaling Pathway

The Hh signaling pathway also plays an important role in maintaining the stemness of CSCs. Additionally, numerous studies demonstrated that deregulation of the Hh pathway plays an important role in tumorigenesis as well as drug resistance in a multitude of cancers by driving cancer cell proliferation, malignancy, metastasis, and the expansion of CSCs. The targeting of this signaling pathway by phytomedicines may inhibit the proliferation and growth of CSCs. Withaferin A, a lactone isolated from the leaf extract of *Withania somnifera,* inhibited the transcriptional activity of the GLI1-DNA complex formed during the Hh signaling pathway in different CSCs. This compound exhibited potent cytotoxicity against PANC1, DU145, and MCF7 cancer cells [75]. One of the popular phytocompounds curcumin, isolated from rhizomes of Turmeric (*Curcuma longa*), was shown to inhibit the Sonic Hh pathway and reduce the expression of breast CSC markers (ALDH1, CD44, OCT4, and CD133). This causes cessation of the cell proliferation and induces the apoptotic cell death in breast CSCs [76]. Furthermore, another constituent, sulforaphane, isolated from cruciferous vegetables including broccoli, has been shown to block the Sonic Hh pathway (Smo, Gli-1, 2) and reduce the markers of EMT (Zeb-1), pluripotency (Oct4, Nanog), angiogenic (VEGF, PDGFRα), and metastasis in pancreatic CSCs. This leads to the induction of apoptosis in pancreatic CSCs, and thus significantly reduced the tumor growth in pancreatic CSC-transplanted NSG mice [18].

One of the polyherbal plant extract preparations, BRM270 (BRMLife), consisting of seven medicinal plants, including *Saururus chinensis, Citrus unshiu Markovich, Aloe vera, Arnebia euchroma, Portulaca oleracea, Prunella vulgaris var. lilacina, and Scutellaria bacicalensis*, has been found to inhibit the metastasis and stemness (SALL4, CD133, Nanog, Sox2, and Oct4) in CD44+ pancreatic ductal adenocarcinoma cells (PDACs) via the Sonic Hh pathway. BRM270 at the dose of 5 mg/kg reduced the tumor growth in a CD44+ PDAC-xenografted mice model [77]. In another study, baicalein, a natural bioactive compound predominantly presents in *Scutellaria bacicalensis* and many other herbal formulations, including QYHJ, was shown to downregulate the pluripotent markers (Sox2, Oct4) and members of the Sonic Hh signaling pathway (SHH, SMO, and Gli-2) in PANC1 CSCs. Baicalein alone at the dose of 60 mg/kg reduced tumor growth in PANC1 CSC-xenografted nude mice. The study results highlighted the therapeutic effect of baicalein against pancreatic CSCs [78]. Another formulation, MSC500, a Korean herbal preparation made up of primarily eight herbs, including *Phellinus linteus, Gastrodiaelata*, and Mulberry leaf, also showed modulation of Hh signaling pathways. This herbal preparation modulated all three signaling pathways (Notch, Wnt, and Sonic Hedgehog) required for the stemness of glioblastoma stem-like cells. MSC500 suppressed the stemness genes as well as CSC markers (Oct4, Sox2, ABCB5, Gli1, Notch1, and β-catenin) in the side population of GBM8401 cancer cells. This results in reduced percentages of side population cells. It has been reported that MSC500 possesses a potent effect against high-grade glioma, and it could be promising for glioma [79].

In addition to their activity on CSCs, phytomedicines have also been found to improve sensitization of CSCs towards the conventional chemotherapeutic drugs. Ovatodiolide, a macrocyclic diterpenoid isolated from *Anisomeles indica* (L.) Kuntze, augmented the chemotherapeutic effect of temozolomide for glioblastoma stem-like cells [26]. It also enhanced the therapeutic effects of cisplatin for nasopharyngeal and oral CSCs [23,24]. Similarly, sulforaphane, commonly found in cruciferous vegetables, augmented the therapeutic effect of cisplatin for nasopharyngeal carcinoma [64]. Curcumin, one of the highly studied natural dietary agents, improved the sensitivity of paclitaxel, cisplatin, doxorubicin, and mitomycin C for breast CSCs [48]. Similarly, ginsenoside-Rb1, a popular compound isolated from *Panax notoginseng,* improved the therapeutic effect of both cisplatin and paclitaxel, commonly used chemotherapeutic drugs for ovarian CSCs [62]. Carnosol, a phenolic diterpene commonly present in Rosemary and Sage, sensitized glioblastoma CSCs to temozolomide for its anti-proliferative effects [57]. Similarly, the combination of aqueous extract of aerial parts of *Gynura divaricata* and cisplatin/doxorubicin/5-Fluorouracil displayed a high level of synergism for treating hepatocellular carcinoma by enhancing cytotoxicity of liver CSCs [20]. These studies demonstrate that phytomedicines not only help in reducing the CSC’s resistance to treatment, but were also shown to synergize the effects of modern chemotherapeutic drugs by improving the sensitivity of cancer cells towards the chemotherapeutic drugs when administered as a combinatorial therapy. Based on the presented studies, more studies are encouraged to investigate the chemo-sensitizing effect of phytomedicines which can be used as adjuvants, and this may help in reducing the dose of modern chemotherapeutic drugs which often cause dose-limiting toxicity, that limit their clinical usage. The targeting of phytomedicinal compounds in the signaling pathways in CSCs has been summarized in Table 3.

## 5. Clinical Studies on Phytomedicines

In recent years, few clinical studies have been carried out to evaluate their safety and efficacy focusing on phytomedicines targeting drug-resistant CSCs and cancer cells. In patients with acute myeloid leukemia, Zhebei granules (formulation of three herbs) combined with chemotherapy have been shown to reduce the percentages of CD34^+^, CD123^+^ and CD33^+^, CD123^+^ leukemia stem cells [81]. The clinical status of some phytomedicines targeting CSCs has been synoptically summarized in Table 4.

## 6. Conclusions and Future Perspectives

In the present review, the recent preclinical and clinical studies of medicinal plants, their bioactive compounds, and herbal preparations shown to be effective against CSCs have been presented. Phytomedicines targeting Hh, Wnt/β-catenin, and Notch signaling pathways as well as the resistance mechanisms involving the CSCs have been summarized using synoptic tables and figures. Targeting of CSCs with phytomedicines show therapeutic promise to reduce the resistance to chemotherapy. The available data are mostly from experimental studies; therefore, additional investigations are necessary to establish the use of phytomedicines in combination with chemotherapeutic agents. Additionally, drug interaction studies are required to understand whether they affect biotransformation and exert the combination showing synergism, antagonism, or additive effects. In most of the studies, the main purified phytoconstituent which could be druggable has been tested for its activities. However, in some studies, the extract of a particular part of a whole plant was tested for its activities. To ascertain the drug discovery and development, the bioactive compounds present in the plant extract need to be characterized, then its mode of action against CSC should be determined. As it is well-established that the plant extracts have numerous bioactive compounds, isolating an individual compound and its mechanism should be encouraged for establishing the role of plant-derived compounds in regulating CSCs. The role of many anticancer agents has been convincingly shown in the experimental studies, and investigating these for targeting CSCs of a particular type of cancer might be more promising from a therapeutic perspective. Furthermore, high-throughput screening of the plant-derived compounds and their synthetic analogues could be useful in order to develop them for pharmaceutical development. There are numerous issues in clinical drug development for the phytomedicines. To name a few, important ones are the physicochemical properties, including poor solubility, stability, and residence time. Various pharmaceutical technologies, including nanoparticle-based delivery, liposomes, and hydrogel formulations, are currently designed to enhance the stability, aqueous solubility, and residence time of the phytomedicines. More research is required to ascertain preclinical and clinical safety as well as the efficacy of the plant-derived bioactive compounds. Studies assessing pharmacokinetic properties along with the pharmacodynamic activity of the phytomedicines will provide a better rationale for the pharmaceutical development of the phytocompounds. Although there is a long way to go to establish these phytomedicines to develop them as drugs targeting CSCs, the phytomedicines shown as efficacious in preclinical studies are promising for future therapeutics targeting CSCs.

## Figures and Tables

**Table 1:** Biomarkers overexpressing on CSCs.

Cancer Type	CSC Markers	References
Ovarian cancer	CD133+/CD44+/CD117+/ALDH1+/ABCG2+	[2]
Stomach cancer	CD44+/CD133+	[8]
Breast cancer	CD44+/ESA+/CD24−/ALDH1+/ABCG2+/EpCAM+/CXCR4	[11]
Leukemia	CD34+CD38-	[16]
Brain tumor	CD133+/CD90+/ALDH1+	[17]
Renal cancer	CD105+	[17]
Pancreatic cancer	CD44+/CD24+/ESA+/CD133+/Bmi1/ALDH1+/ABCG2+/CXCR4	[18]
Colon cancer	CD133+/ALDH1+/CD44+/EpCAM	[19]
Liver cancer	CD133+/CD90+/CD44+/ABCG2+/EpCAM+/CD13+	[20]
Prostate cancer	CD44+/CD133+/ALDH1+/Bmi1	[21]
Lung cancer	CD133+/CD117+/ALDH1+/ABCG2+/EpCAM	[22]
Nasopharyngeal cancer	CD44+/CD133+/ALDH1+/ABCG2+/Bmi1	[23]
Oral cancer	CD44+/ALDH1+/CD117+/Bmi1	[24]
Melanoma	ABCB5+/ALDH1+/CD133+/CD44+/CD117+	[25]
Glioblastoma	CD133+/CD44+/Bmi1	[26]

ABCB5: ATP-Binding Cassette Sub-family B Member 5; ABCG2: ATP-Binding Cassette Sub-family G Member 2; ALDH1: Aldehyde Dehydrogenase 1A1; Bmi1: B cell-Specific Moloney Murine Leukemia Virus Integration Site 1; CD24: Heat-Stable Antigen; CD34: Hematopoietic Progenitor Cell Antigen; CD38: Cyclic ADP Ribose Hydrolase; CD44: Hyaluronate Receptor; CD90: Thymocyte Differentiation Antigen-1; CD133: Prominin-1; CD117: c-kit; CXCR4: Chemokine Receptor; EpCAM: Epithelial Cell Adhesion Molecule; ESA: epithelial surface antigen.

**Table 2:** Phytomedicinal compounds targeting the key regulators of anti-cancer drug resistance in CSCs.

Plant Source	Extract	Bioactive Compound	Mode of Action	In Vivo Dose	Cells/Model	References
*Alstonia scholaris*	Fruit extract	Scholarisine Q(1) and R(2)	Induction of apoptosis	------	Glioma stem cells	[40]
*Anisomeles indica*	------	Ovatodiolide	Induction of apoptosis Modulation of EMT process Downregulation of CD44, CD133, Sox2, and Oct4 Dysregulation of JAK2/STAT3 pathway	------	Glioblastoma stem-like cells	[26]
			Induction of apoptosis Downregulation of Sox2 and Oct4 Increase of E-cadherin Dysregulation of JAK2/STAT3 pathway	------	CSC population in nasopharyngeal carcinoma	[23]
			Induction of apoptosis Downregulation of CD133, Klf4, Oct4, Nanog, and JARID1B Dysregulation of JAK2/STAT3 pathway	3.6 mg/kg	Oral CSCs and xenograft tumor mice	[24]
Cruciferous vegetables	------	Phenethyl isothiocyanate	Downregulation of Oct4, Nanog, and Sox2	20 mg/kg	Colon CSCs and xenograft tumor mice	[41]
			Promotion of oxidative stress Suppression of Sp1 transcription factor	10 µM	Cervical CSCs and Xenograft NOD-SCID tumor mice	[42]
*Atractylodes macrocephala Koidz*	Rhizome extract	Atractylenolide I	Downregulation of the phosphorylation of proteins related to the AKT/mTOR pathway Alteration of apoptosis, glucose metabolism, and stem-like behavior	25 mg/kg and 75 mg/kg	Stemness of colon cancer cells and xenograft tumor mice	[43]
*Fructus viticis*	------	Flavonoids	Decrease the phosphorylation level of Akt Downregulation of CD133, CD44, and ALDH1, Bmi1, Nanog, and Oct4, Twist1, and Snail1	------	Lung CSCs	[44]
Pigeon pea	------	Cajaninstilbene acid derivatives	Cytotoxic (pathway not deduced)	------	Breast cancer stem-like cells	[45]
*Berberis libanotica Ehrenb*	Root extract	------	G0–G1 arrest Inhibition of cellular migration and sphere formation	------	Prostate CSCs	[46]
*Berberis*, *Arcangelisia Hydrastis*	------	Berberine	G0/G1 cell cycle arrest Cancer stemness inhibition (attenuation of CD133, β-catenin, n-myc, Sox2, Notch2, and nestin) EMT reversal by downregulation of PI3/Akt and Ras-Raf-ERK signaling.	------	Stemness in neuroblastoma cells	[32]
*Dendrobium* *venustum*	Stem extract	Lusianthridin	Downregulation of Src-STAT3-c-Myc pathways Pro-survival suppression and pro-apoptotic induction Abolishment of stemness (decrease in CD133, ABCG2, and ALDH1A1)	------	Lung CSCs	[22]
*Curcuma longa*	------	Curcumin	Downregulation of expression of Vimentin, Fibronectin, β-catenin, and upregulation of E-cadherin Decreased expression of Sox2, Nanog, and Oct4	------	Breast CSCs	[47]
	------	Curcumin	Reduction in the expression of ABC transporters ABCG2 and ABCC1	------	Breast CSCs	[48]
	------	Curcumin	Induction of apoptosis Dysregulation of JAK/STAT3 signaling pathway	------	Papillary thyroid CSCs	[49]
	------	Curcumin	Induction of apoptosis	5 mg/kg	Glioblastoma stem cells and xenograft tumor mice	[50]
*Walsura pinnata Hassk*	Bark extract	Betulonic acid	Induction of intrinsic apoptosis	18, 36, or 54 μM	Leukemia stem cells and xenotransplanted zebrafish	[16]
*Costus speciosus*	Rhizome extract	------	Induction of apoptosis G0/G1 and G2/M arrest	------	Stemness of prostate cancer cells	[51]
*Viola odorata*	Hydro-alcoholic extract of aerial part	------	Induction of apoptosis	------	Breast CSCs	[52]
*Polygonum cuspidatum*	Root extract	2-Ethoxystypandrone	Induction of apoptosis Inhibition of STAT3 signaling	------	Hepatocellular CSCs	[53]
*Cinnamomum cassia*	------	Cinnamic acid	Downregulation of CSC-associated markers (OCT4, NANOG, ABCB1, and ALDH1A) and the proportion of CSCs (SP cells, CD44- and CD133-positive cells)	------	Colon CSCs	[19]
*Glycyrrhiza glabra*	------	Glabridin	Epigenetic regulation of miR-148a/SMAD2 signaling	20 mg/kg	Breast cancer stem-like cells and xenograft tumor mice	[54]
*Morus australis*	------	Morusin	Induction of apoptosis Attenuation of NF-κB activity	------	Cervical CSCs	[55]
*Lithospermum erythrorhizon*	------	Shikonin	Involvement of JNK/c-Jun pathway	2 mg/kg	Glioblastoma stem cells and xenograft tumor mice	[56]
*Rosmarinus officinalis*	------	Carnosol	Induction of apoptosis via p53 functional reactivation Modulation of EMT	------	Glioblastoma CSCs	[57]
PienTze Huang	------	------	Inhibition of ABCB1 and ABCG2	------	Colorectal CSCs	[58]
*Allium sativum*	------	Allicin (diallyl thiosulfinate)	Increased expression of *cyclin D1*	------	Melanoma stem-like cells	[25]

------ = NA.

**Table 3:** Phytomedicinal compounds targeting Wnt/β-catenin, Notch, and Sonic Hedgehog signaling pathways in CSCs.

Plant Source	Extract	Bioactive Compound	Mode of Action	In Vivo Dose	Cell Line/Model	References
*Abrus precatorius*	Seed extract	Abrus agglutinin	p73 suppressed Snail expression, leading to EMT inhibition Induction of intrinsic and extrinsic apoptosis Inactivation of Wnt/β-catenin signaling pathway	50 μg/kg	Oral squamous carcinoma stem-like cells and xenograft tumor mice	[63]
Celandine (TCM)	------	Sanguinarine	Downregulation of Wnt/β-catenin signaling pathway	0.5 mg/20 g	Lung CSCs and xenograft tumor mice	[67]
*Gynura divaricata*	Aqueous extract of aerial part	------	Downregulation of Wnt/β-catenin signaling pathway	300 mg/kg	Hepatocellular CSCs and xenograft tumor mice	[20]
*Panax quinquefolius*	------	Ginsenoside-Rb1	Inhibition of Wnt/β-catenin signaling Inhibition of EMT	50 mg/kg	Ovarian CSCs and xenograft tumor mice	[62]
*Allium sativum*	------	Diallyl-trisulfide	Induction of apoptosis Modulation of Wnt/β-catenin signaling pathway	------	Colorectal CSCs	[61]
*Murraya koenigii* *(L) Spreng*	Leaf extract	Koenimbin	Induction of apoptosis by intrinsic pathway Downregulation of Wnt/β-catenin self-renewal pathway	------	Breast CSCs	[21]
			Induction of apoptosis via intrinsic pathway G0/G1 phase arrest	------	Prostate CSCs	[80]
*Chelidonium majus/Macleaya cordata*	------	Chelerythrine chloride	Downregulation of β-catenin	------	Non-small cell lung carcinoma stem-like cells	[66]
*Evodiae rutaecarpa*	------	Evodiamine	Inhibition of Wnt Signaling	------	Gastric CSCs	[70]
*Evodiae fructus*	------	Evodiamine	Induction of apoptosis Suppression of Notch and Wnt Signaling	------	Colon CSCs	[69]
Baizuan (TCM)	------	Gomisin M2	Downregulation of Wnt/β-catenin signaling pathway Induction of apoptosis	10 μM	Breast CSCs and zebrafish xenograft	[68]
*Cruciferous* *vegetables*	------	Sulforaphane	Downregulation of DNA methyltransferase1 Restoring the expression of Wnt inhibitory factor 1	60 mg/kg	Nasopharyngeal CSCs and xenograft tumor mice	[64]
			Suppression of miR-19 and Wnt/β-catenin pathway	------	Lung CSCs	[65]
			Modulation of Sonic hedgehog–Gli pathway Inhibition of pluripotency markers, angiogenesis markers, and EMT markers	------	Pancreatic CSCs	[18]
*Curcuma longa*	------	Curcumin	Suppression of Sonic Hedgehog pathway Induction of apoptosis Decreased expression of CSC markers	------	Bladder CSCs	[50]
*Withania somnifera*	Leaf extract	Withaferin A	Hh signal inhibition	------	Pancreatic, prostate, and breast CSCs	[75]
BRM270	Alcohol extract	------	Suppression of Sonic Hedgehog pathway Induction of apoptosis	5 mg/kg	Pancreatic ductal adenocarcinoma stem cells and xenograft tumor mice	[77]
Qingyihuaji (TCM)	------	Baicalein	Modulation of Sonic Hedgehog pathway	20 or 60 mg/kg	Pancreatic CSCs and xenograft tumor mice	[78]
Qingyihuaji (TCM)	Aqueous extract	------	Downregulation of Notch-4 and Jagged-1 in Notch signaling pathway	36 g/kg	Pancreatic cancer stem-like cells and xenograft tumor mice	[71]
Xiaotansanjie (TCM)	------	------	Inhibition of Notch-1	1.46, 2.92, and 5.84 g/mL	Gastric CSCs and xenograft tumor mice	[72]
*Psoralea corylifolia*	------	Psoralidin	Inhibition of Notch-1 signaling Inhibition of EMT	------	Breast CSCs	[74]
PienTze Huang (TCM)	------	------	Induction of apoptosis Suppression of Notch-1 signaling pathway	------	Colorectal CSCs	[73]
MSC500	------	------	Suppression of ALDH, ABCB5, Oct4, Sox2, β-catenin, Gli-1, and Notch-1	------	Glioblastoma stem cells	[79]

------ = NA.

**Table 4:** Clinical status of phytomedicinal compounds targeting CSCs.

Clinical Trial No.	Sponsors and Collaborators	Title of the Study	Clinical Status	Year of Study (Start Date–Completion Date)
Cruciferous Vegetable/Sulforaphane				
NCT00982319	Sidney Kimmel Comprehensive Cancer Center at Johns Hopkins	Study to Evaluate the Effect of Sulforaphane in Broccoli Sprout Extract on Breast Tissue	Phase 2	2009–2013
NCT03665922	University of Pittsburgh	Biomarkers of Sulforaphane/Broccoli Sprout Extract in Prostate Cancer	Recruiting (Phase not applicable)	2019–2024
2. Curcumin				
NCT01740323	Andrew H Miller and National Cancer Institute	Phase II Study of Curcumin vs. Placebo for Chemotherapy-Treated Breast Cancer Patients Undergoing Radiotherapy	Phase 2	2015–2018
NCT03980509	Medical University of South Carolina	A “Window Trial” on Curcumin for Invasive Breast Cancer Primary Tumors	Phase 1	2020–2021
NCT03072992	National Center of Oncology, Armenia, and BRIU GmbH	“Curcumin” in Combination with Chemotherapy in Advanced Breast Cancer	Phase 2	2017–2019
3. Cruciferous Vegetable/Phenethyl isothiocyanate (PEITC)				
NCT01790204	Georgetown University	A Study of the Effects of PEITC on Oral Cells with Mutant p53	Phase 2	2012–2014
NCT00691132	University of Minnesota and National Cancer Institute	Phenethyl Isothiocyanate in Preventing Lung Cancer in Smokers	Phase 2	2009–2013
4. Garlic				
NCT00079170	National Cancer Institute	Docetaxel Plus Garlic in Treating Patients with Locally Advanced or Metastatic Breast Cancer	Pilot study (Phase not applicable)	2004–2007
5. Berberine				
NCT02226185	Shanghai Jiao Tong University School of Medicine	Study of Berberine Hydrochloride in Prevention of Colorectal Adenomas Recurrence	Phase 3	2014–2018
6. Licorice				
NCT00176631	Rutgers, The State University of New Jersey, and National Cancer Institute	Licorice Root Extract and Docetaxel in Treating Patients with Metastatic Prostate Cancer That Did Not Respond to Hormone Therapy	Phase 2	2007–2008
7. N-butylidenephthalide				
NCT03234595	Everfront Biotech Co., Ltd.	A Phase I/IIa Study of Cerebraca Wafer Plus Adjuvant Temozolomide (TMZ) in Patients with Recurrent High-Grade Glioma	Phase 2	2017–2021
8. Ginsenoside				
NCT02714608	Tasly Pharmaceuticals, Inc.	A Study of Ginsenoside H Dripping Pills for Advanced Non-Small Cell Lung Cancer (NSCLC)	Phase 2	2016–2018
9. Ashwagandha				
NCT00689195	Tata Memorial Hospital and Pharmanza Herbals Pvt Limited (PHPL)	Pilot Study of Curcumin Formulation and Ashwagandha Extract in Advanced Osteosarcoma (OSCAT)	Phase 2	2008–2013

## Data Availability

Not Applicable.
